# COVID-19 Solutions Are Climate Solutions: Lessons From Reusable Gowns

**DOI:** 10.3389/fpubh.2020.590275

**Published:** 2020-11-25

**Authors:** Natalie Baker, Rebecca Bromley-Dulfano, Joshua Chan, Anshal Gupta, Luciana Herman, Navami Jain, Anita Lowe Taylor, Jonathan Lu, Jaspreet Pannu, Lisa Patel, Mary Prunicki

**Affiliations:** ^1^Stanford University, Stanford, CA, United States; ^2^Stanford University School of Medicine, Stanford, CA, United States; ^3^Stanford Law School, Stanford, CA, United States; ^4^Department of Physical Medicine and Rehabilitation, Stanford University School of Medicine, Stanford, CA, United States; ^5^Department of Internal Medicine, Stanford University School of Medicine, Stanford, CA, United States; ^6^Department of Pediatrics, Stanford University School of Medicine, Stanford, CA, United States; ^7^Sean N. Parker Center for Allergy and Asthma Research, Stanford University, Stanford, CA, United States

**Keywords:** reusable gown, isolation gown, climate-smart healthcare, safety, resilience, sustainability, COVID-19, PPE

## Abstract

The COVID-19 pandemic has laid bare the inadequacy of the U.S. healthcare system to deliver timely and resilient care. According to the American Hospital Association, the pandemic has created a $202 billion loss across the healthcare industry, forcing health care systems to lay off workers and making hospitals scramble to minimize supply chain costs. However, as the demand for personal protective equipment (PPE) grows, hospitals have sacrificed sustainable solutions for disposable options that, although convenient, will exacerbate supply strains, financial burden, and waste. We advocate for reusable gowns as a means to lower health care costs, address climate change, and improve resilience while preserving the safety of health care workers. Reusable gowns' polyester material provides comparable capacity to reduce microbial cross-transmission and liquid penetration. In addition, previous hospitals have reported a 50% cost reduction in gown expenditures after adopting reusable gowns; given the current 2000% price increase in isolation gowns during COVID-19, reusable gown use will build both healthcare resilience and security from price fluctuations. Finally, with the United States' medical waste stream worsening, reusable isolation gowns show promising reductions in energy and water use, solid waste, and carbon footprint. The gowns are shown to withstand laundering 75–100 times in contrast to the single-use disposable gown. The circumstances of the pandemic forewarn the need to shift our single-use PPE practices to standardized reusable applications. Ultimately, sustainable forms of protective equipment can help us prepare for future crises that challenge the resilience of the healthcare system.

## Introduction

In the spring of 2020, the COVID-19 pandemic swept across the United States and overwhelmed the supply of personal protective equipment. Countries around the world saw images of US nurses wearing garbage bags due to gown shortages (1). Nurses and doctors were given one mask for multiple patient encounters, which both increased their risk of infection and the risk of infecting others (2). A pandemic of this nature has long been predicted given our expedited intrusion into wildlife habitats and exposure to pathogens (3). Lessons from building healthcare resiliency during COVID-19 could hold the key for dealing with another, perhaps greater, health crisis: climate change.

Fossil fuel pollution from healthcare harms patient health. Air pollution from the healthcare industry is estimated to cause 405,000 lost disability-adjusted life years in the U.S. every year (4). Preventing the worst impacts of climate change requires achieving net-zero greenhouse gas emissions by 2050 (5). The U.S. healthcare sector alone is responsible for 10% of U.S. greenhouse gas emissions, 64% of which comes from supply chain (4); therefore, averting the worst effects of climate change requires substantial supply chain emissions reductions (6).

Climate-smart healthcare is healthcare that is both low-carbon and builds resilience to climate change (3). Here, we provide one example of how COVID-19 solutions can be climate solutions: a review of the literature surrounding reusable isolation gowns, the second-most-used piece of personal protective equipment (PPE) following gloves (7).

Johns Hopkins estimated that a single 100-day COVID-19 wave would require an additional 321,000,000 isolation gowns on top of baseline isolation gown use in hospital inpatients, emergency departments, emergency medical services, outpatient visits, and nursing homes in the U.S. (8). As this model assumed strict social distancing, even more PPE may be needed if and when compliance decreases (9). Prominent successful deployments of reusable gowns at institutions like the Ronald Reagan UCLA Medical Center and Carilion Clinic in Roanoke, Virginia, have demonstrated how reusable gowns are safer, cheaper, and more sustainable than disposable gowns. Reusable PPE is especially relevant during the pandemic, as it ensures supply stability given increased demand. Finally, replacement of disposable isolation gowns with reusable isolation gowns demonstrated a 28% reduction in energy consumption, a 30% reduction in greenhouse gas emissions, a 41% reduction in blue water consumption, and a 93% reduction in solid waste generation (10).

Although reusable gowns have clear benefits, 80% of U.S. hospitals currently use disposable isolation gowns (7). In this literature review, we use MeSH terms and keywords to index PubMed articles and catalog websites and procurement guides. We find that reusable isolation gowns are poised as an excellent first step for hospitals to save money, stay safe, and transition to climate-smart healthcare practices.

## Safety

The purpose of PPE is to protect wearers from the spread of infectious diseases. The type and need for an isolation gown depend on the anticipated amount of contact with potentially infectious material. This is reflected in two classification systems for protective apparel safety, the Association for the Advancement of Medical Instrumentation (AAMI) and the Occupational Safety and Health Administration (OSHA) (11, 12). Although many reusable and disposable isolation gowns on the market are OSHA-compliant and follow the AAMI criteria, we now assess whether certain textiles are more effective barriers than others.

Reusable isolation gowns are typically composed of polyester, but several are composed of cotton or a blend of both fabrics (13). Synthetic fibers such as polypropylene and polyester have been shown to exhibit less liquid penetration compared to natural fibers such as cotton (14). Loosely woven cotton gowns, which were common historically, have since been pulled from the market due to high permeability (15). Reusable gown cuffs are typically knitted; however, limited research exists comparing the safety of knitted cuffs to the woven and nonwoven designs in disposable gowns. To reinforce their strength and further minimize the risk of cross-transmission, reusable gowns can be treated with repellant and antibacterial finishes. Studies have shown that these treatments may reduce the risk of bacterial cross-transmission and microorganism penetration, even when gowns show no visible liquid penetration (7). Research into new, eco-friendly antimicrobial finishes such as chitosan or peroxy acids is ongoing (15). Reusable gowns can typically withstand 75–100 washes while retaining maximum repellency. Laundry services can track the number of washes using marked grids or add chlorofluorocarbon to the gown wash to ensure reusability (16).

Disposable gowns are typically composed of synthetic fibers such as polypropylene, polyester, and polyethylene. They are designed with nonwoven processes which utilize either thermal, chemical, or mechanical fiber-bonding; meanwhile, reusable gowns tend to be woven. Although the random order of fibers in nonwoven fabrics has been shown to limit penetration by liquids, there is high variability in gown production, and no existing literature shows nonwoven fabrics to be safer than impermeable woven fabrics, especially woven polyester (T280) (15, 17, 18). The comparable safety provided by reusable gowns is especially important as the demand for gowns soars during and potentially after the global pandemic.

The CDC recommends a shift toward reusable isolation gowns composed of polyester and polyester-cotton fabrics (19).

## Cost

The pandemic has created enormous financial burdens on hospitals and health systems with an estimated loss of $202 billion across the industry (20). With these losses, health care systems have already started to furlough and lay off health care workers. Solutions that can decrease the cost of the pandemic for hospital systems could help relieve this impact.

Case studies demonstrate that a transition to reusable isolation gowns can result in significant cost savings. For example, the Ronald Reagan UCLA Medical Center has saved over $1.1 million in 3 years after implementing 3.3 million reusable gowns (see Case Studies section for more details) (21). In another case study, the Carilion Clinic, a healthcare system encompassing over 195 hospitals and clinics saved over $850,000 over 3 years after transitioning to reusable isolation gowns (22). After comparing the net investment costs with incremental savings, Carilion Clinic calculated a return on investment (ROI) period of only 6 months in a pre-COVID world. They estimated a cost savings of nearly 50% per gown use, with $0.79 per use for disposable compared to $0.39 per use for reusable gowns. Though few peer-reviewed studies have been published on the cost-savings of reusable isolation gowns, myriad studies have found similar cost-savings or cost-equivalence in the context of surgical gowns (23–26).

One study found that a single 100-day COVID-19 wave would require an additional 321,000,000 isolation gowns in the U.S. healthcare system (8). Assuming proportional cost savings to those for Carilion Clinic, reusable gown use would save healthcare systems an estimated $128,400,000 in surge gowns over that 100-day period alone. These savings may be greater during large-scale crises when competition over limited single-use supplies drive up costs. Researchers at the Society for Healthcare Organization Procurement Professionals found that the COVID-19 pandemic has led to a 2000% price increase for isolation gowns, from $0.25 to $5 per gown (27). With new disposable gowns required for every provider and every provider requiring multiple gowns per day, the demand and therefore cost of gowns has skyrocketed. Reusable gowns provide not only a baseline cost savings but also price and supply stability during times of high PPE demand.

## Environmental Sustainability

Reusable isolation gowns offer a tremendous opportunity to reduce environmental impact across their lifetime. Life cycle inventory (LCI) studies consistently find that, while initial manufacturing of reusable gowns might be more energy-intensive than disposable gowns over their lifetime, reusable isolation gowns use less energy, produce less waste, and generate less greenhouse gas emissions compared to disposable gowns (10, 15, 28, 29).

**Figure 1:**
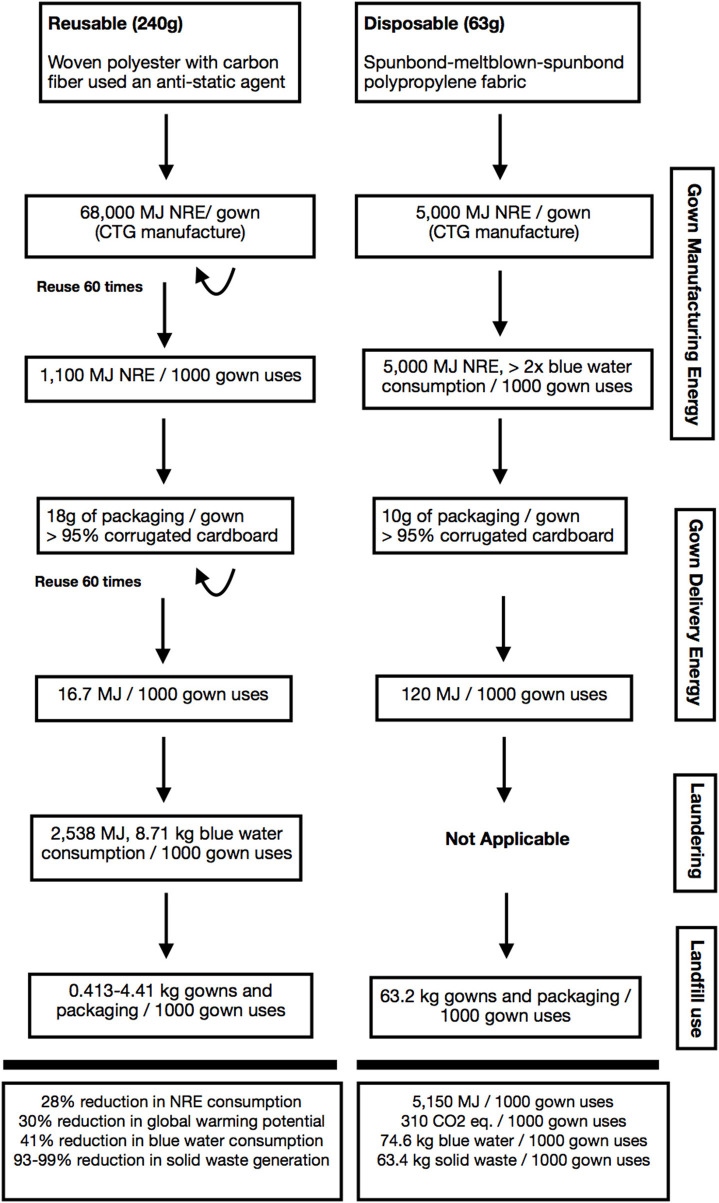
Life cycle inventory of reusable vs. single-use isolation gowns. Environmental costs of each step of a life cycle inventory (LCI) as reported in Vozzola et al. (10). *Environmental considerations in the selection of isolation gowns: A life cycle assessment of reusable and disposable alternatives*. While reusable gowns exhibit higher footprints in manufacturing and delivery energy expenditure, re-calculated footprints based on gown use reveal significant reductions across all four categories of environmental indicators. Vertical boxes on the right indicate stage of life cycle inventory. CO_2_ eq, carbon dioxide equivalent; MJ, megajoules; NRE, natural resource energy.

In a systematic evaluation of isolation gowns that included the impacts of manufacturing, packaging, and landfill disposal of disposable gowns compared to reusable gowns, reusables were found to consume 28% less total energy over the cradle-to-grave product life cycle (10). In addition, reusable gown use led to a 30% reduction in greenhouse gas emissions and a 93–99% reduction in solid waste generation at the studied healthcare facility (Figure 1). Finally, blue water consumption in reusable gown systems was found to be half the consumption level typical to disposable gown systems (10).

A Life Cycle Inventory (LCI) of reusable vs. disposable isolation gowns, commissioned by the Textile Rental Services Association of America, found that even the best case scenario of disposable gowns (polypropylene, spunbond nonwoven with more efficient manufacturing and transportation) had higher global warming potential than the worst case for reusable gowns (polyethylene with less efficient manufacturing, transportation, and washing) at 61 kg CO_2_ compared with 41 kg CO_2_, respectively. In addition, the best case of disposable gowns had substantially higher acidification potential, eutrophication potential, smog creation potential, and primary energy demand, and similar-to-higher ozone depletion potential, compared to the worst case of reusable gowns. The impacts of disposable gowns were primarily linked to raw materials (polypropylene) and manufacturing, while impacts of reusable gowns were dominated by washing (29).

A cradle-to-use comparison of reusable medical patient gown (55% cotton/45% polyester with a halamine antimicrobial surface) to a disposable gown (polypropylene Spunbond-Meltblown-Spunbond fabric) found that the reusable gown consumes 71% less energy than the disposable gown (65,049 MJ compared to 225,947 MJ, respectively, per 75,000 gown uses), assuming 75 reuses per reusable gown. In addition, the reusable gown produces significantly less air, water and solid chemical emissions. The reusable gown uses fewer raw materials overall, excluding water. Even assuming only ten reuses, the reusable gown achieves lower energy usage than the disposable gown (28).

We emphasize that the results must be contextualized based on the unique fabric (e.g., 100% cotton vs. cotton/polyester mix), manufacturing process, and laundering process of the gowns, as well as the antimicrobial finish and application process. For example, nanoscale Silver (nAg) is an attractive antimicrobial product used in many consumer textiles. A comparison of nAg enabling processes found substantial variation in environmental impacts (30). Environmental impacts from silver must be addressed, for example, through textile wastewater treatment (31).

Acknowledging the relative scarcity of studies on isolation gowns, we compared our findings to the literature on surgical gowns and drapes in the OR. A recent cradle-to-end-of-life analysis, including natural resources, creation, use and reuse, laundering, sterilization, and transportation, and end-of-life disposal of reusable surgical gowns against disposables found that using reusable gowns reduced natural resource energy consumption by 64%, greenhouse gas emissions by 66%, blue water consumption by 83%, and solid waste generation by 84% when compared with disposable gowns (32). This is consistent with earlier studies of reusable vs. disposable surgical textiles: across six large life-cycle studies, researchers found that, compared with reusable textiles, disposable textiles require 200–300% more energy and 250–330% more water, generate 750% more solid waste and generate a 200–300% larger carbon footprint (24).

## Case Studies

Reusable isolation gowns have been in widespread use at many medical sites, including Carilion Clinic and Ronald Reagan UCLA Medical Center.

Carilion Clinic has been using reusable isolation gowns since 2011, when clinics experienced gown quotas from the H1N1 outbreak. Short gown supply, combined with dissatisfaction over the waste from disposables, drove the project's initiation. Reusable gowns were assessed based on clinical performance (i.e., safety), cost, environmental impact, and user experience. Gowns were designed and deployed in a manner that received full approval from the infection control committee, and several safety measures were taken with each use. First, gowns were inspected for stains and tears before repackaging. Second, for each inspection, a quality control grid on the isolation gown was marked with a symbol unique to the employee, which allowed both tracking of the gown's useful lifetime as well as tracing of any quality control problems. Finally, the isolation gown was re-rinsed with a barrier re-treatment product (22).

Carilion Clinic has now been using reusable gowns for over 9 years. They found that the deployment addressed all considerations from infection control and resulted in a savings of $851,984 over the initial 3 years. This deployment also eliminated 514,839 pounds of waste, and end users appreciated the increased comfort, coverage, barrier protection, storage space, and decreased environmental impact (22). Notably, during the COVID-19 pandemic, Carilion Clinic did not experience any isolation gown supply disruptions (33).

The UCLA reusable isolation gown pilot project began in 2012 and was aimed at reducing waste. Reusable gowns were designed through a multi-stakeholder effort including vendors and infection control staff. Reusable gowns were piloted at the liver transplant unit, which had the highest turnover of 1,000 disposable gowns used per day. While some staff members voiced concerns on gown comfort and washing with patient linen, these concerns dissipated over time with education efforts and steady cultural shift. Following the pilot study, the medical center introduced reusable gowns on a unit by unit basis, achieving full conversion over a 4-year period. More than 3.3 million reusable gowns have been used since 2012, saving over $1.1 million. Tracking reusable gown lifespan is currently done by inspection, but the institution is considering more formal tracking by RFID-type scanners. Over a 3-year period (2011–2015), this program diverted 297 tons of waste from the landfill (34).

## Discussion

We have discussed how reusable gowns are safer, more cost-effective, and more sustainable than disposable gowns. Still, reusable gowns remain widely unused due to several concerns. The first is safety: although reusable gowns are available in several layers of protection, all of which meet or exceed the AAMI safety standards (35), institutions may still worry that reusable gowns could lose protective capacity with repeated laundering (13). Several studies have shown this concern can be addressed by adding layers to aid in repellency. Ronald Reagan UCLA Medical Center has found their reusable isolation gowns to have a lifespan of 75–100 washes (21). Carilion Clinic's reusable isolation gowns had higher coverage and protection than their isolation gowns, in part due to their barrier retreatment product (22). Thus, the protective capacity of reusable gowns can be addressed and need not be a barrier to adoption.

Another potential barrier is patient and staff thermal discomfort with reusable isolation gowns compared to disposable alternatives (13). At UCLA, initial complaints that the gowns were uncomfortably hot eventually dissipated as staff became accustomed to wearing reusable gowns (21). A separate study found that the type of gown (disposable or reusable) played little to no role in patient and staff compliance (36). Another reported that reusable gowns engineered using microfiber technology and 100% polyester material both met protection standards and were more comfortable than disposable gowns (35).

Successful transition to reusable gowns requires both initiative and consideration of institutional needs. A proper workflow includes engagement of all stakeholders, especially infection control, and education on the superior protective properties of reusables. Practice Greenhealth provides an example workflow to adopt reusable gowns (tailored for surgical gowns, but easily applicable to isolation gowns) (37). This can be used to quickly track new deployment.

A key determinant of reusable gown adoption is market forces, especially affecting cost and supply. For example, during the pandemic, UCSF substantially increased its use of reusable gowns in response to the increased demand and shorter supply for disposable gowns. Carilion Clinic made their initial switch to reusables during the H1N1 outbreak to achieve a robust supply of gowns (22). However, these supply strains of disposable gowns are not confirmed by quantitative analysis; further research is warranted to evaluate whether increased reusable isolation gown use provides supply chain stability that would not otherwise be provided by disposable isolation gowns.

## Future Directions

Winter is approaching, and with it an expected spike of infectious disease cases like COVID-19. At the same time, steady offshoring of PPE to other countries has left the U.S. vulnerable to supply chain disruptions (38). Countries like Spain and India also suffered severe PPE shortages that placed frontline healthcare workers at risk (39). To adequately protect healthcare providers, we must address key vulnerabilities in our PPE supply chain and current practices.

First, we advocate for increased funding for multidisciplinary, clinically translatable research on sustainable PPE practices. Though isolation gowns are among the better studied items of reusable PPE, shortages of masks and face shields must also be addressed. At present, there are only two groups in the U.S. that we are aware of who are actively pursuing research in this area, with one studying the efficacy, supply, environmental impact, and usage of reusable PPE and the other developing biocidal air filters for reusable PPE (40, 41). Both groups are funded by the NSF RAPID grant. Researchers outside of the U.S. are similarly investigating the feasibility and/or impacts of transitioning to reusable materials in light of pandemic-driven PPE shortages particularly as they related to readily accessible materials and ensuring reliability in the reusable PPE supply chain (42–44). While these individual examples are important, more dedicated research funding toward reusable PPE is needed to support prompt translation for healthcare systems during this urgent time. Specifically, further research is needed to understand how public policy can incentivize sustainable PPE adoption and facilitate healthcare system transitions at scale. Research and deployment should engage all relevant stakeholders, including end users (healthcare workers), vendors, infection control, and linen services. Open data on sustainable PPE should be published and collected, similar to how the N95DECON consortium has gathered resources for decontamination of N95 masks (45). Public-private partnerships can further support reusable PPE deployment; Hanes and other clothing brands have already been working with FEMA to increase reusable PPE production (46).

Second, we call for the rapid adoption of evidence-based sustainable PPE into clinical practice across the U.S. This will require buy-in by hospital leaders, infection control, departmental advocates and supply chain; as well as concurrent public policy to incentivize sustainable PPE. Our presented data on cost and safety, together with case studies from medical centers who have successfully used reusable isolation gowns for years, should address common concerns about reusable PPE. Policymakers can aid by ensuring a robust supply chain for reusable PPE, designing incentives for reusable PPE production and usage, and educating staff toward transitioning to reusable PPE practices.

## Author Contributions

All authors listed have made a substantial, direct and intellectual contribution to the work, and approved it for publication.

## Conflict of Interest

The authors declare that the research was conducted in the absence of any commercial or financial relationships that could be construed as a potential conflict of interest.
